# Quindoline-derivatives display potent G-quadruplex-mediated antiviral activity against herpes simplex virus 1

**DOI:** 10.1016/j.antiviral.2022.105432

**Published:** 2022-12

**Authors:** Ilaria Frasson, Paola Soldà, Matteo Nadai, Martina Tassinari, Matteo Scalabrin, Vijay Gokhale, Laurence H. Hurley, Sara N. Richter

**Affiliations:** a Department of Molecular Medicine, University of Padua, Padua, Italy; b College of Pharmacy, University of Arizona, Tucson, AZ, 85721, United States; c BIO5 Institute, University of Arizona, Tucson, AZ, 85721, United States

**Keywords:** HSV-1, G-quadruplex, Antiviral activity, Quindoline derivatives, ICP4, CC_50_, Half Maximal Cytotoxicity, G4, G-quadruplex, hpi, hours post infection, HSV-1, Herpes Simplex Virus type 1, IC_50_, Half Maximal Inhibitory Concentration, ICP4, Infected Cell Polypetide 4, MOI, Multiplicity Of Infection

## Abstract

G-quadruplexes (G4s) are non-canonical nucleic acid structures that regulate key biological processes, from transcription to genome replication both in humans and viruses. The herpes simplex virus-1 (HSV-1) genome is prone to form G4s that, along with proteins, regulate its viral cycle. General G4 ligands have been shown to hamper the viral cycle, pointing to viral G4s as original antiviral targets. Because cellular G4s are also normally present in infected cells, the quest for improved anti-HSV-1 G4 ligands is still open. Here, we evaluated a series of new quindoline-derivatives which showed high binding to and stabilization of the viral G4s. They displayed nanomolar-range anti-HSV-1 activity paralleled by negligible cytotoxicity in human cells, thus proving remarkable selectivity. The best-in-class compound inhibited the viral life cycle at the early times post infection up to the step of viral genome replication. In infected human cells, it reduced expression of ICP4, the main viral transcription factor, by stabilizing the G4s embedded in ICP4 promoter. Quindoline-derivatives thus emerge as a new class of G4 ligands with potent dual anti HSV-1 activity.

## Highlights

• A novel class of quindoline-derivatives displayed potent binding to and stabilization of HSV-1 G-quadruplexes (G4s).
• Quindoline-derivatives have outstanding antiviral activity coupled to negligible cytotoxicity. SAR is provided.
• The best-in-class inhibited viral genome replication and expression of the major transcription factor by G4 stabilization.

## Graphical abstract

**Figure:**
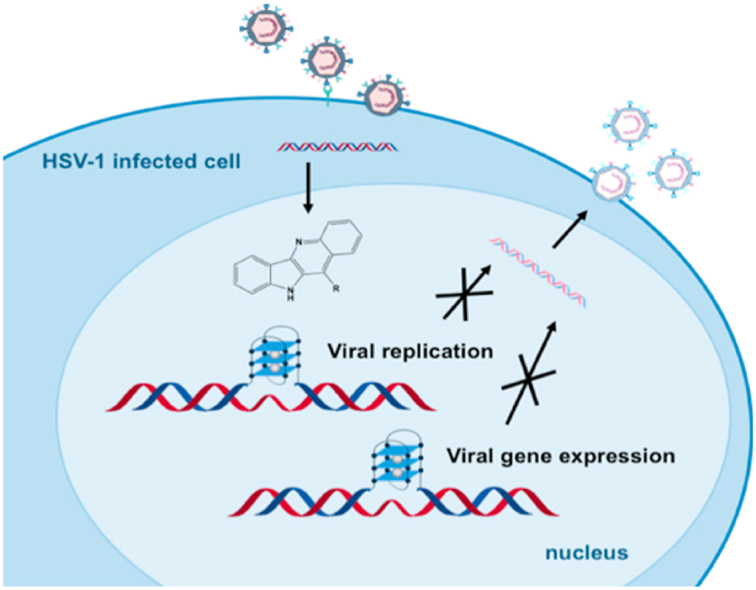

## 1 Introduction

Guanine quadruplexes (G4s) are non-canonical nucleic acids structures, which are being been widely investigated for their unique structural and functional features (Spiegel et al., 2020). G4s form in G-rich DNA strands: four guanine residues joined through Hoogsteen hydrogen bonds constitute a G-tetrad; G4s form when two or more G-tetrads stack on top of each other, coordinated by cations. Physiological cations, such as K^+^ and Na^+^, are the most relevant G-tetrads stabilizers. G4s display different conformations (i.e. parallel, antiparallel or mixed), which depend on the spatial orientation of adjacent guanine tracts, as well as on the length and nucleotide composition of connecting nucleotides, which are referred to as loops (Bryan and Baumann, 2011; Burge et al., 2006; Pipier et al., 2021).

G4s play pivotal roles at the human genome level (Hänsel-Hertsch et al., 2017), acting as switchers of crucial cellular processes such as transcription, replication, DNA repair and others (Varshney et al., 2020). More recently, G4s have been predicted and reported in the genome of microorganisms (Saranathan and Vivekanandan, 2019). In particular, G4s exert key functions in numerous DNA and RNA viruses infecting humans and at different stages of the viral life cycle (Lavezzo et al., 2018; Ruggiero et al., 2021; Ruggiero and Richter, 2018). Indeed, targeting viral G4s for antiviral purposes has been proposed and validated *in vitro* against several viruses (Ruggiero et al., 2021; Ruggiero and Richter, 2018).

The Herpes Simplex Virus 1 (HSV-1) infection is largely distributed in the worldwide adult population (∼80%), being associated with both oral/genital sores as well as herpetic blindness and meningoencephalitis (“STD Facts - Genital Herpes,” 2020; World Health Organization, 2022). HSV-1 infection has also been involved in the onset of the Alzheimer disease (Cairns et al., 2022; Eimer et al., 2018; Itzhaki et al., 2020; Marcocci et al., 2020; Piacentini et al., 2014; Rizzo, 2020; Shinomoto et al., 2021). Treatments are based exclusively on nucleoside analog-based drugs, such as acyclovir and its derivatives (Seley-Radtke and Yates, 2018): they are efficient and display low toxicity, but resistance traits are rapidly emerging (Chen et al., 2021). Thus, development of novel drug-like molecules, with new mechanisms of action against HSV-1 infection are urgently needed. Multiple G4 clusters are present in the HSV-1 genome, both in repeat regions and in promoter of immediate early genes that control the viral life cycle; HSV-1 G4s also mediate the association of viral proteins to the viral genome (Artusi et al., 2015, 2016; Frasson et al., 2019, 2021). G4-ligands displayed anti-HSV-1 activity based on inhibition of viral replication (Artusi et al., 2015; Callegaro et al., 2017).

To search for improved anti-HSV-1 activity, we here tested a novel class of Quindoline-derived compounds. Quindoline is a derivative of the natural product cryptolepine, which binds and stabilizes G4s in the c-myc promoter (Boddupally et al., 2012; Dai et al., 2011; Dickerhoff et al., 2021; Ou et al., 2011). We evaluated their ability to selectively inhibit the HSV-1 viral cycle. Compound GSA-0932 showed remarkable antiviral properties and negligible toxicity in human host cells. It drastically reduced transcription of ICP4, the major viral transcription factor, through stabilization of the G4s embedded in its own promoter, while concurrently, inhibiting viral DNA replication, through stabilization of the many G4s embedded in the repeat regions of the HSV-1 genome.

## 2 Materials and methods

The complete description of this section is available in Supplementary Information.

## 3 Results

### 3.1 Chemistry

**Scheme 1:**
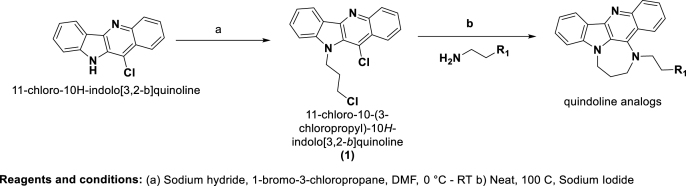
Synthesis of quindoline analogs.

GQC-05, an ellipticine derivative was identified as G4 binding agent (Brown et al., 2011). GSA-0820 is a quindoline derivative and was synthesized from the common intermediate 11-chloroindoloquinoline 1 (Table 1) (Boddupally et al., 2012; Miranti et al., 2020). GSA-0932 and related analogs were developed with an aim to disrupt planarity of the parent quindoline and impart novel structural features that would increase the binding to G4s (Table 2). These compounds were synthesized starting from 11-chloroindoloquinoline using two step synthesis (Scheme 1). One of the major features in the synthesis is the cyclization step to create seven membered cyclic system in the quindoline structure. Different analogs carrying a variety of side chains with varying properties were synthesized and tested.

**Table 1:** Structures of compounds used in the study.

Compound	Structure	Chemical Name
Quindoline		*N*^1^,*N*^1^-diethyl-*N*^2^-(10*H*-indolo[3,2-*b*]quinolin-11-yl)ethane-1,2-diamine
GQC-05		2-((5,11-dimethyl-6*H*-pyrido[4,3-*b*]carbazol-9-yl)oxy)-*N*,*N*-dimethylethan-1-amine
GSA-0820		2-(4-(10*H*-indolo[3,2-*b*]quinolin-11-yl)piperazin-1-yl)-*N*,*N*-dimethylethan-1-amine

**Table 2:**
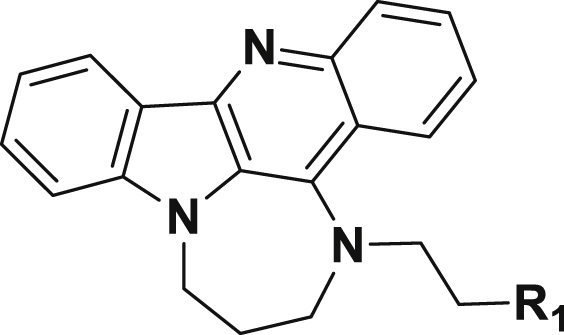
Structures of quindoline analogs used in the study.

Compound	R_1_	Chemical Name
GSA-0825		4-(3-(2,3-dihydro-4,9,13b-triazabenzo[*b*]cyclohepta[*lm*]fluoren-4(1*H*)-yl)propyl)morpholine
GSA-0903		4-(3-(4-methylpiperazin-1-yl)propyl)-1,2,3,4-tetrahydro-4,9,13b-triazabenzo[*b*]cyclohepta[*lm*]fluorene
GSA-0920		4-(3-(azepan-1-yl)propyl)-1,2,3,4-tetrahydro-4,9,13b-triazabenzo[*b*]cyclohepta[*lm*]fluorene
GSA-0932		1-(2-(2,3-dihydro-4,9,13b-triazabenzo[*b*]cyclohepta[*lm*]fluoren-4(1*H*)-yl)ethyl)piperidin-4-ol
GSA-1202		4-(3-(piperidin-1-yl)propyl)-1,2,3,4-tetrahydro-4,9,13b-triazabenzo[*b*]cyclohepta[*lm*]fluorene
GSA-1502		2-(2,3-dihydro-4,9,13b-triazabenzo[*b*]cyclohepta[*lm*]fluoren-4(1*H*)-yl)-*N*,*N*-dimethylethan-1-amine
GSA-1504		3-(2,3-dihydro-4,9,13b-triazabenzo[*b*]cyclohepta[*lm*]fluoren-4(1*H*)-yl)-*N*,*N*-dimethylpropan-1-amine
GSA-1512		4-(2-(2,3-dihydro-4,9,13b-triazabenzo[*b*]cyclohepta[*lm*]fluoren-4(1*H*)-yl)ethyl)cyclohexan-1-ol

### 3.2 Quindoline-derived compounds display significant anti-HSV-1 activity

**Fig. 1:**
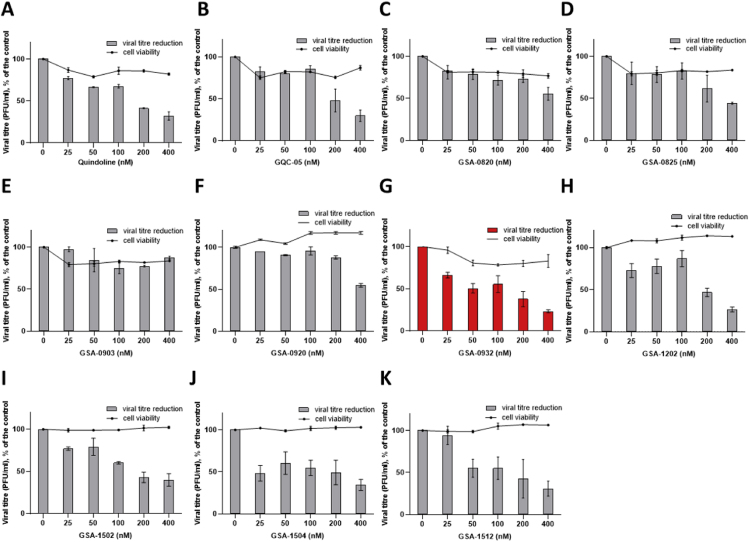
**Antiviral activity of Quindoline and derivatives in HSV-1-infected U-2 OS cells. Infected cells were treated with increasing concentrations of each compound and newly produced infective viral particles were quantified by PRA. Antiviral activity is expressed as plaque forming units per milliliter (PFU/ml), calculated over the viral titre of vehicle treated cells. Cell viability was measured by MTT assay on uninfected cells treated with the same compound concentration used in the antiviral assay. Viral titre reduction (bars) and cell viability (line) at each tested concentration are reported. Mean values of two independent experiments, with three replicates per condition, and SD are reported**.

We initially tested quindoline-derivatives, and quindoline as parent compound, for their antiproliferative activity against the human U-2 OS cell line, which is permissive to HSV-1 infection (Deschamps and Kalamvoki, 2017; Frasson et al., 2021). The compounds' cytotoxic effect was calculated as the concentration that reduced U-2 OS cells viability by 50%, with respect to the vehicle-treated cells, and was expressed as cytotoxic concentration (CC_50_, Table 3, Fig. S1). Antiviral activity was assessed and reported as inhibitory concentration (IC_50_), i.e. the compound concentration able to reduce the viral titre by 50% with respect to the vehicle-treated infected cells (Table 3). The antiviral assays were performed at compounds’ concentrations that displayed less than 20% cytotoxic activity on host cells. For each compound, both CC_50_ values and cell viability curves in the presence of increasing concentrations of compounds were reported (Table 3 and Fig. 1A–K). The selectivity index (SI) of each compound was calculated as the ratio of CC_50_ over IC_50_ values (Table 3): the higher the SI value, the more effective and less toxic the antiviral drug (U.S. Food and Drug Administration, 2006). The viral titre reduction obtained at the highest tested concentration (400 nM) was also reported (Table 3).

**Table 3:** **Anti-HSV-1 activity and cytotoxicity of the Quindoline-derived compounds.** Summary table reporting the tested compound antiviral properties, indicated as Inhibitory Concentration 50 (IC_50_, compound concentration required to inhibit 50% of HSV-1 viral titre), in relation to the Cytotoxic Concentration 50 (CC_50_, compound concentration able to reduce host cell viability by 50%). The ratio between the IC_50_ and the CC_50_ is the Selectivity Index (SI).

Compound	U-2 OS cell line			
	IC_50_ (nM)	CC_50_ (nM)	SI	Viral titre reduction (%) at the highest tested concentration
**Quindoline**	155.9 ± 6.7	2200.2 ± 489.3	14.1	68
**GQC-05**	267.4 ± 21.8	1654.6 ± 118.5	6.2	72
**GSA-0820**	>400	2984.8 ± 171.2	<7.5	45
**GSA-0825**	356.9 ± 60.9	32709.2 ± 313.8	91.6	56
**GSA-0903**	>400	5205.8 ± 492.8	<13.0	12.5
**GSA-0920**	>400	3620.6 ± 92.1	9.1	45
**GSA-0932**	165.0 ± 49.1	19395.3 ± 738.0	117.5	77
**GSA-1202**	248.0 ± 64.4	4852.9 ± 261.3	19.6	73
**GSA-1502**	155.49 ± 27.6	14758.5 ± 1067.4	95.2	60
**GSA-1504**	308.7 ± 82.2	4509.6 ± 516.8	14.6	65
**GSA-1512**	255.6 ± 114.3	18089.5 ± 389.8	70.8	69

Most compounds displayed considerable antiviral activity, with IC_50_ values in the nanomolar range. The parent compound quindoline, GSA-0932 and GSA-1502 displayed the highest anti HSV-1 activity, with similar inhibitory concentrations (∼160 nM) (Table 3 and Fig. 1A, G, I). GQC-05, GSA-0932 and GSA-1202 induced viral inhibition higher than 70% at the highest tested concentration (Table 3 and Fig. 1B, G, H). GSA-0820, GSA-0825, GSA-0903, and GSA-0920 displayed low cytotoxicity coupled to insufficient antiviral activity, suggesting low cell permeability to the four compounds (Table 3 and Fig. 1C-F). Some compounds, as GSA-1202 and GSA-1504, did not display a strict dose-dependent response curve, possibly due to aggregation issues, and thus were not considered further. GSA-0932 displayed the highest SI, coupled to the highest reduction of viral yield at the highest tested concentration (77%, Table 3) and was thus selected for further investigation.

### 3.3 Quindoline derivative GSA-0932 selectively stabilizes HSV-1 G4s

**Fig. 2:**
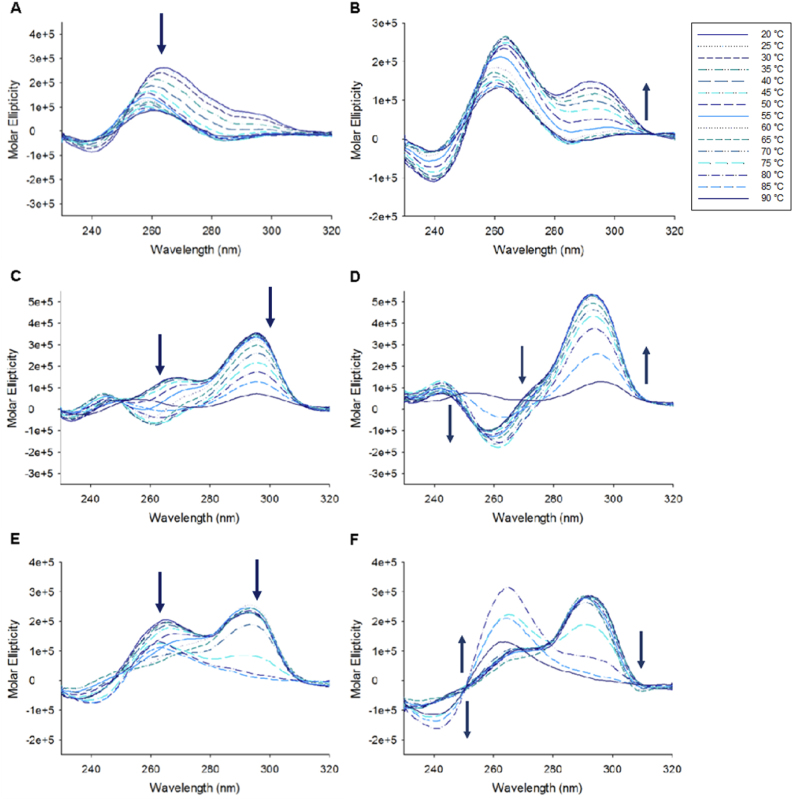
**Thermal unfolding on the HSV-1 G4 sequences folded in 2.5 mM KCl in the absence and presence of GSA-0932.** (**A**) *un3* G4 alone and (**B**) with GSA-0932; (**C**) *un2* G4 alone and (**D**) with GSA-0932; (**E**) *gp054a* G4 alone and (**F**) with GSA-0932. Spectra were recorded over a temperature range of 20–90 °C. Oligonucleotide folding was tested in two independent assays, one replicate per condition. Representative spectra per each oligonucleotide are shown. Arrows indicate the direction of changes in molar ellipticity.

To characterize GSA-0932 binding and selectivity towards HSV-1-1 G4s, circular dichroism thermal stability analysis was performed. Both conformational and stability changes were taken into consideration. Three representative and previously characterized HSV-1 G4s were used: *un2* is embedded in the terminal repeat of the HSV-1 genome, *gp054a* resides in the coding region of the *gp054* gene, which encodes for the essential tegument protein UL36, *un3* is embedded in the promoter of the Infected Cell Polypeptide 34.5 (ICP34.5) gene (Artusi et al., 2015; Frasson et al., 2021). Viral G4s were analyzed by CD spectroscopy in the presence of physiological (100 mM) or lower K^+^ concentration (2.5 mM) to best appreciate compound effect over extremely stable G4 structures (e.g., *un2* sequence). The topology and thermal stability of each sequence was analyzed in the absence and presence of GSA-0932 (Fig. 2, Table 4, Figs. S2–S4).

**Table 4:** **Melting temperatures (*T***_***m***_**) of HSV-1 G4 sequences in the absence/presence of GSA-0932.***T*_*m*_ values (°C) were calculated according to the van ’t Hoff equation. KCl concentrations are indicated. SD indicates standard deviation, nd stands for “not determined”.

	2.5 mM K^+^		100 mM K^+^	
	*T*_*m*_ (°C)	Δ*T*_*m*_ ± SD (°C)	*T*_*m*_ (°C)	Δ*T*_*m*_ ± SD (°C)
*un3*	37.6 ± 0.4		65.0	
*un3* + GSA-0932	56.4 ± 1.2	18.8 ± 0.8	66.4	1.4
*un2*	82.4 ± 0.3		>90	
*un2* + GSA-0932	>90	>7.5	>90	nd
*gp054a*	57.3/82.4		58.9/>90	
*gp054a* + GSA-0932	74.7/>90	17.4/>7.5	74.5/>90	15.6

All sequences displayed hybrid topologies with positive peaks at 260 nm and 290 nm and a negative peak at 240 nm (Fig. 2A, C and E, and Table 4) at all tested K^+^ concentrations. Upon addition of the compound, a general increase in the CD signal was observed (Fig. 2B, D and F). All HSV-1 G4s were stabilized by the compound (Table 4).

Selectivity of GSA-0932 towards HSV-1 G4s was next investigated by competition electrospray ionization mass spectrometry (ESI-MS). We had previously shown that ESI/MS conditions induce negligible effects on G4 topology and stability (Scalabrin et al., 2017). The HSV-1 G4s were analyzed in the presence of hTel or c-myc G4s, as cellular competitors. (Table 5).

**Table 5:** **Relative binding affinity analyzed by MS competition assay for *un3*, *un2*, *gp054a*, *hTel*, and *c-myc* G4 oligonucleotides.** nd stands for not determined (due to the technical impossibility to correctly assign MS peaks to the respective samples).

Competing G4s	*un3*	*un2*	*gp054a*	Cell G4 (*hTel* or *c-myc*)
*un3*/*hTel*	50.3 ± 1.2			13.8 ± 0.17
*un3*/*c-myc*	37.1 ± 0.77			59.5 ± 1.1
*un2*/*hTel*		38.9 ± 1.70		11.1 ± 0.36
*un2*/*c-myc*		29.6 ± 1.9		46.2 ± 1.59
*gp054a*/*hTel*			57.3 ± 0.43	nd
*gp054a*/*c-myc*			51.8 ± 0.42	45.6 ± 0.75

MS analysis showed that GSA-0932 preferentially binds the viral G4s *un2*, *un3*, and *gp054a* over the telomeric G4s sequence (*hTel*). The compound showed high binding affinity also towards c-myc G4, in line with the reported affinity of Quindoline (GSA-0932 parental backbone) towards it (Brooks and Hurley, 2010; Brown et al., 2011; Dai et al., 2011).

**Fig. 3:**
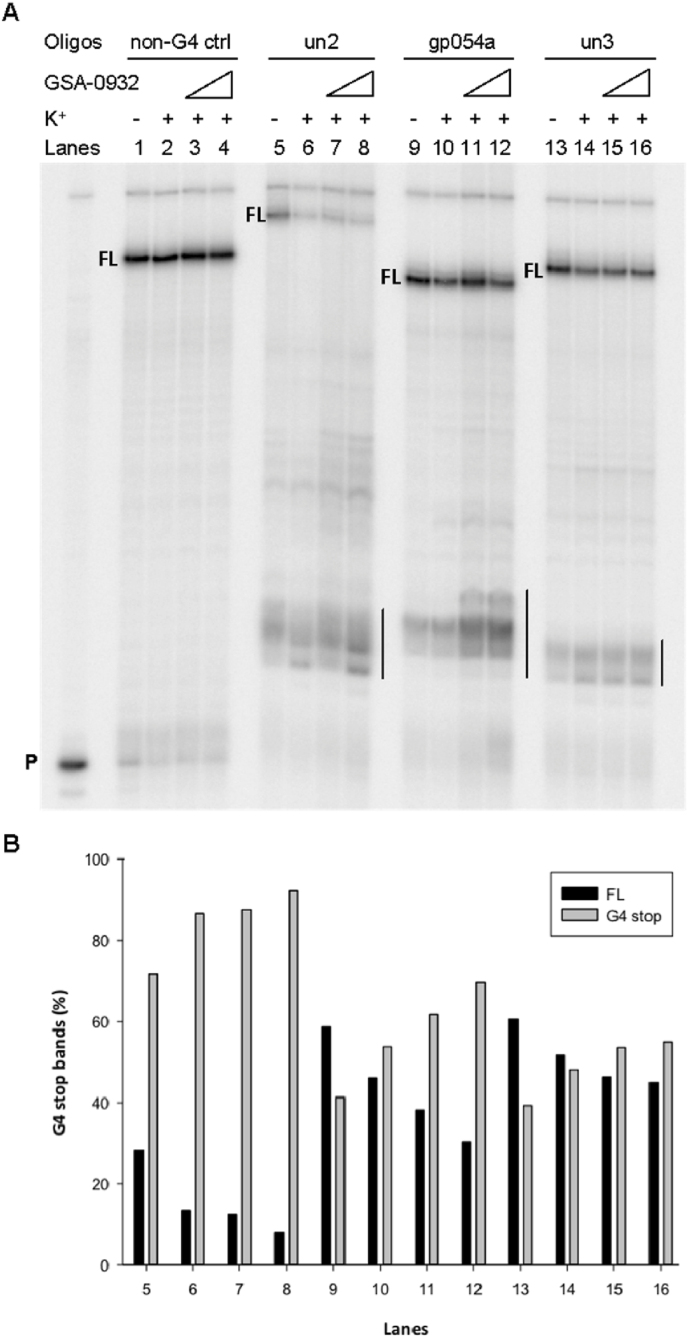
***Taq*****polymerase stop assay on HSV-1 G4s.** (**A**) *Un2*, *gp054a, un3* templates were amplified by *Taq* polymerase in the absence (lanes 5,9,13) and presence of K^+^, combined with increasing amounts (2–8 μM) of GSA-0932 (lanes 7–8, 11–12, 15–16) or the same amount of DMSO as that in the ligand (lanes 6,10,14). *Un3* was analyzed at 50 mM of K^+^, while the other sequences were investigated at 0.5 mM of K^+^. A template (non-G4 ctrl) unable to fold into G4 was also used as control (lanes 1–4). P stands for unreacted labeled primer, FL stands for full-length product. G4-specific *Taq* polymerase stop sites are highlighted by vertical bars. (**B**) Full-length and G4 stop bands intensity quantification relative to the *Taq* polymerase stop assay shown in panel (A).

The effect of GSA-0932 stabilization at HSV-1 G4s on *Taq* polymerase processivity was assessed on *un2*, *un3* and *gp054a* forming sequences and a sequence unable to fold into G4 as negative control. In the absence of K^+^ (Fig. 3A, lanes 5, 9, 13), all three G4-forming sequences already displayed visible stop sites (Fig. 3A, lanes 5, 9, 13), confirming G4 folding in the K^+^-containing polymerase buffer (∼50 mM). In the presence of higher K^+^ concentrations (Fig. 3A, lanes 6-10-14) G4-mediated stop sites slightly increased in all templates, indicating that the additional K^+^ further stimulated G4 folding and consequent polymerase stalling. *Un3* was analyzed at 50 mM of K^+^, while un2 and gp054a were investigated at 0.5 mM of K^+^ due to their high stability. Addition of increasing concentrations (2–8 μM) of GSA-0932 induced concentration-dependent increment of G4-induced polymerase stalling (Fig. 3A, lanes 5, 9, 13, vertical lines). Reduction of full-length amplicons was alongside visible (Fig. 3B). In contrast, GSA-0932 had no effect on the elongation of the negative control DNA template, confirming the G4-dependency of the observed polymerase inhibition.

These results showed the ability of GSA-0932 to bind and stabilize several HSV-1 G4s, which differ in terms of topology and stability. GSA-0932 bound the c-myc G4 with slightly higher affinity, when compared to the tested viral G4s, nonetheless HSV-1 G4s were preferred over the telomeric G4s, which represent the most abundant cellular G4s in infected human cells. The compound induced Taq polymerase stalling, implying that GSA-0932 antiviral effect was induced by a block of DNA processivity enzymes at the abundant viral G4s (Artusi et al., 2015, 2016, 2021; Callegaro et al., 2017; Frasson et al., 2019, 2021).

### 3.4 GSA-0932 acts in early events of the viral life cycle

**Fig. 4:**
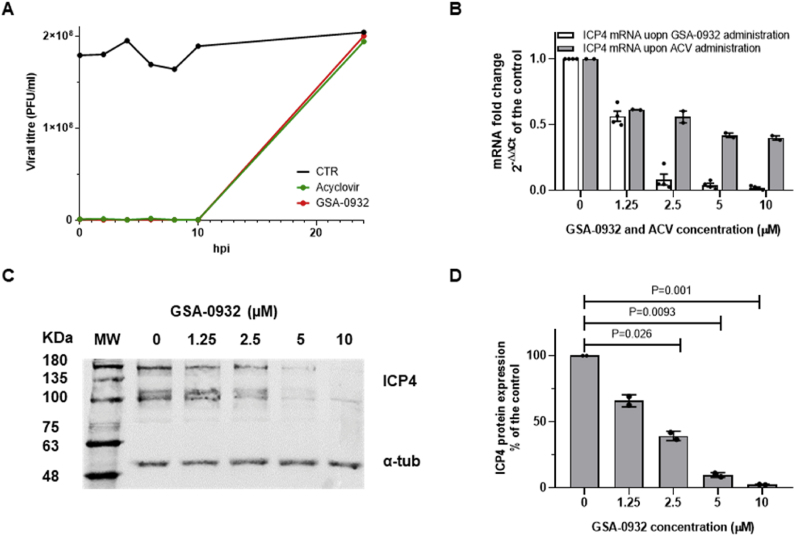
**GSA-0932 hinders viral genome replication and strongly affects ICP4 expression levels in infected cells.** (**A**) Time-of-addition of GSA-0932 in U-2 OS cells infected with HSV-1 (MOI 1). GSA-0932 (12.4 μM, 75-fold its IC_50_) was added at different times (0, 2, 4, 6, 8, 10 h) post infection (Red line). Acyclovir was used as a reference drug (45 μM, 75-fold its IC_50_, green line). Vehicle-treated cells were used as control (black line,CTR). (**B**) U-2 OS cells were infected with HSV-1 (MOI 1) and treated with increasing concentrations of GSA-0932 or ACV (0–10 μM). Total RNA was extracted from infected cells at 6 hpi and mRNA expression levels of ICP4 were measured. ACV was used as the reference antiviral drug for HSV-1. (**C**) Western Blot analysis of ICP4 expression upon GSA-0932 treatment of HSV-1 infected U-2 OS cells (6 hpi, 0–10 μM). The alpha-tubulin protein was used as housekeeping control gene (**D**) Western Blot quantification of ICP4 levels normalized to the housekeeping protein. Data are mean ± s.e.m. of n = 2 biological replicates. Each condition was tested in duplicate per replicate. S.e.m. stands for standard error of the mean.

To assess GSA-0932 mechanism of action within the viral cycle, we set up a Time-of-addition (TOA) assay, where the highest compound concentration unable to induce host cell toxicity was administrated to infected cells at different times post-infection (Fig. 4A). TOA assay consents to highlight the last viral process targeted by a test compound by comparing its activity to reference antiviral drugs (Daelemans et al., 2011). In our assay, the reference drug was acyclovir (ACV), a thoroughly characterized inhibitor of HSV-1 DNA polymerase (Elion, 1983; Gnann et al., 1983; Taylor and Gerriets, 2021). U-2 OS cells were infected (MOI 1) and treated with GSA-0932 (12.375 μM) or ACV (45 μM), corresponding to 75-fold their calculated/reported IC_50_ values for strain F HSV-1 (Crute et al., 2002; Daelemans et al., 2011), the highest possible concentration devoid of cytotoxicity (Chu et al., 2020; Honda et al., 2001). Compounds were added from T0 (time of infection) up to T10 (10 h post infection, hpi), by 2 h intervals.

ACV-treatment of HSV-1-infected U-2 OS cells showed pronounced viral titre reduction when the compound was administrated up to 8 hpi, whereas increasing viral titres were detected afterwards. This is in line with the reported ACV mechanism of action, as the drug targets viral DNA polymerase during viral DNA replication that occurs between 6 and 12 hpi (Ibáñez et al., 2018; Weller and Coen, 2012). GSA-0932 hindered viral cycle progression when administrated up to 8 hpi. Up to 6 hpi, GSA-0932 blocked HSV-1 infection more potently than ACV. These data suggest that GSA-0932 inhibits viral replication similarly to ACV (Everett, 2014), and also that GSA-0932 could act at earlier stages. Indeed, key regulatory G4s in the promoters of the immediate early viral genes have recently been reported (Frasson et al., 2019, 2021).

HSV-1 IE genes are necessary to prime the host cells for viral gene expression: among them, the major HSV-1 transcription factor, ICP4, regulates viral transcription via G4 binding and unfolding, and it concurrently regulates its own expression recognizing the G4s embedded in its own promoter (Frasson et al., 2021). We thus tested GSA-0932 for its ability to downregulate ICP4 expression via G4 stabilization. To test this hypothesis, we first checked GSA-0932 binding to the four G4s embedded in the ICP4 promoter (Figs. S5–S6 and Table S2). U-2 OS infected cells (MOI 1) were treated with GSA-0932 or ACV at increasing concentrations (0–10 μM), ICP4 expression levels (mRNA copies) were evaluated at 6 hpi (Fig. 4B), hence at the time of the highest ICP4 expression and prior to massive viral replication, (Dremel and DeLuca, 2019). ICP4 transcription levels were normalized by the human TATA-binding protein (TBP) expression level (Radonić et al., 2005). Both GSA-0932 and ACV reduced ICP4 expression at all the tested concentrations but, compared to ACV, GSA-0932 was remarkably more potent, as it almost completely abolished ICP4 transcription at the highest tested concentrations (2.5, 5 and 10 μM). ICP4 protein levels were assessed by Western Blot analysis in the same conditions (Fig. 4C). GSA-0932 induced significant dose-dependent reduction of ICP4 expression that exceeded 95% at the highest compound concentration (Fig. 4D). These data indicate that GSA-0932 inhibits the HSV-1 viral cycle by also acting at early stages, i.e. hampering the expression of ICP4, the major viral IE protein.

To further characterize GSA-0932 antiviral activity, its distribution in the nucleus of HSV-1- and mock-infected cells was examined. First, GSA-0932 UV–visible and fluorescence spectra were recorded (Fig. S7). The compound displayed absorption peaks at 419, 346, 332, and 286 nm, and a main emission peak at 458 nm (Fig. S4), which allowed its detection in human cells by diode laser. GSA-0932 widely distributed in the nucleus of mock-infected cells upon 2 h treatment at augmenting concentrations (4–8 μM) (Fig. S8A–C). Punctate aggregations of GSA-0932 were visible in some cells, which may correspond to nucleoli and nucleolin accumulation (Miranti et al., 2020). No difference in compound cellular distribution was detected at the tested concentrations.

**Fig. 5:**
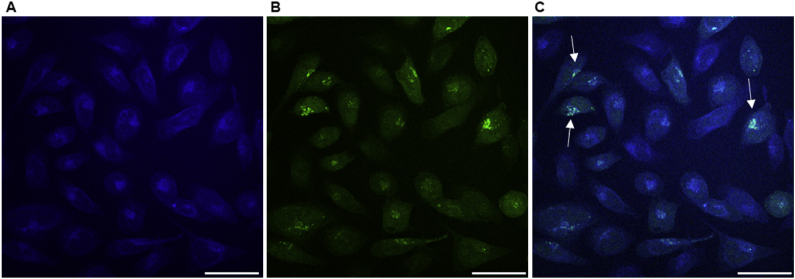
**Colocalization between GSA-0932 and the recombinant VP16-GFP virus.** U-2 OS cells were seeded on coverslips, infected with the recombinant HSV-1, and treated for 2 h with the tested compound. The 8 hpi time point is shown. (**A**) GSA-0932 (6 μM) signal in blue; (**B**) VP16-GFP HSV-1 signal in green; (**C**) merge of the two signals. Arrows indicate colocalization areas. White lines are scale bars (50 μm).

To study GSA-0932 cellular distribution during HSV-1 infection, a recombinant HSV-1 expressing the green fluorescent protein (GFP) fused to the viral protein VP16 (HSV-1 [V41]) was used (GFP-HSV-1). GFP HSV-1 shows the same time-regulated replication cycle of the wild-type virus (La Boissière et al., 2004). U-2 OS cells were infected with GFP-HSV-1 (MOI 3) and treated with GSA-0932. At 8 hpi the newly synthetized VP16-GFP reached the nucleus of infected cells and remarkable nuclear foci due to VP16 accumulation at the HSV-1 viral DNA replication sites were observed (Fig. 5B) (Artusi et al., 2016, 2021; La Boissière et al., 2004). In infected cells, GSA-0932 signal distributed in the nucleus (Fig. 5A) with distinctive clusters, which co-localized with HSV-1 VP16 protein, thus indicating that the compound accumulates in the typical nuclear areas where the new viral genomes are produced (Fig. 5A–C) (Artusi et al., 2016, 2021; La Boissière et al., 2004).

These data indicate that GSA-0932 efficiently hampers HSV-1 replication by hindering the viral cycle up to the replication steps, with an initial G4-mediated downregulation of the major IE genes, followed by accumulation in the nuclear viral replication compartments with subsequent impediment of viral DNA replication.

## 4 Discussion

In the present work, we tested quindoline-derived G4 ligands as a novel class of G4 ligands for their ability to selectively target HSV-1 G4s (Artusi et al., 2015; Frasson et al., 2019, 2021; Lavezzo et al., 2018). Conventional G4-ligands are often characterized by large aromatic scaffolds with reported administration and selectivity issues (Balasubramanian et al., 2011; Ruggiero and Richter, 2018). The drug-likeness of the quindoline-derived G4 ligands and their reduced cytotoxicity in human cells lines make them attractive molecules for antiviral purposes (Boddupally et al., 2012; Ou et al., 2011). Indeed, most of the tested quindoline-derivatives displayed significant antiviral activity against HSV-1, in the nanomolar range of concentrations. Most candidates exhibited promising selectivity indexes, due to negligible cytotoxicity in human host cells. Some compounds (i.e. GSA-1202 and GSA-1504) did not display a strict dose-dependent response curve, possibly due to aggregation issues, and thus were not further considered. GSA-0932 offered the best combination of potent anti-HSV-1 activity and high selectivity (SI). Both activity and selectivity data on GSA-0932 analogs show interesting structure-activity relationship (SAR) trends. i) Compounds with two carbon linkers between seven-membered ring and R_1_ group (GSA-0932, GSA-1502, GSA-1512) display higher SI values. ii) Compounds with three carbon linkers (GSA-0903, GSA-0920, GSA-1202 and GSA-1504) show lower SI values. iii) GSA-0932 consists of both positively charged basic nitrogen and hydrogen bonding group (-OH) for its interaction with phosphate backbone of quadruplex structure.

GSA-0932 displayed excellent affinity for the viral G4s and it hampered HSV-1 viral cycle acting up to the viral replication step. Since we have previously observed that IE gene expression is modulated by multiple G4s embedded in IE gene promoters (Artusi et al., 2015, 2016; Frasson et al., 2019, 2021), we investigated and observed remarkable activity of GSA-0932 on the expression of ICP4, HSV-1 main transcription factor, at the early stages of viral infection. In contrast, ACV displayed only minor effect on ICP4, thus confirming that ACV acts on viral genome replication (Liu et al., 2021; Shen et al., 2019). Hence, GSA-0932 by acting at G4s both reduced HSV-1 genome replication, similarly to other G4 ligands (Artusi et al., 2015, 2021; Callegaro et al., 2017) and acyclovir (Elion, 1983), and restricted ICP4 gene/protein expression.

GSA-0932 was able to bind and stabilize different G4s: it showed remarkable affinity towards HSV-1 structures but maintained good binding also to c-myc G4 (King et al., 2020; Miranti et al., 2020), while displaying low affinity for telomeric G4s. In infected cells, viral G4s rapidly overcome the amount of cell G4s, fact that may explain the highly selective antiviral activity of GSA-0932 (Artusi et al., 2015, 2016; Frasson et al., 2019, 2021). However, binding of GSA-0932 to c-myc G4 might also improve its antiviral activity: it has been reported that HSV-1 modulates c-myc expression in infected cells (Alfonso-Dunn et al., 2017; Birkenheuer et al., 2018; Hilton et al., 1995; Hu et al., 2016; MacLeod and Minson, 2010; Polpitiya Arachchige et al., 2018), to likely augment cell permissiveness to the virus. GSA-0932 binding to c-myc G4 drops oncogene transcription in human cells (Hilton et al., 1995), so that GSA-0932 may affect HSV-1 replication in infected cells both by hindering viral transcription and replication through direct interaction at viral G4s and by disrupting the cellular response prompted by the virus to allow its replication.

## 5 Conclusions

The described data on quindoline-derivatives confirmed the crucial role played by G4s in the HSV-1 viral cycle and pointed out how G4 ligands can be used as effective and innovative tools in the management of viral infections. The possibility that G4 ligands recognize both the viral target G4s and cellular G4s which help increase the antiviral activity, will allow the development of innovative antiviral strategies that will lead to novel cutting-edge therapeutic approaches in the treatment of crucial human viral-related diseases.

## Declaration of competing interest

The authors declare that they have no known competing financial interests or personal relationships that could have appeared to influence the work reported in this paper.

## Data Availability

Data will be made available on request.
