# Supplementary material for: Quindoline-derivatives display potent G-quadruplex-mediated antiviral activity against herpes simplex virus 1
Source: Antiviral Res. 2022 Dec;208:105432. doi: 10.1016/j.antiviral.2022.105432 (PMC9720158; doi:10.1016/j.antiviral.2022.105432)
Supplement: Multimedia component 1 [file mmc1.pdf]

Quindoline-derivatives display potent G-quadruplex-mediated antiviral activity against herpes simplex virus 1

Ilaria Frasson,[1] Paola Soldà,[1] Matteo Nadai,[1] Martina Tassinari,[1] Matteo Scalabrin,[1] Vijay Gokhale,[3] Laurence H. Hurley[2] and Sara N. Richter[1]*

[1]Department of Molecular Medicine, University of Padua, Padua, Italy

[2]College of Pharmacy, University of Arizona, Tucson, Arizona 85721, United States.

[3]BIO5 Institute, University of Arizona, Tucson, Arizona 85721, United States

SUPPLEMENTARY DATA

2. Materials and methods

2.1 Synthesis

For synthesis of compounds GSA-0825, GSA-0903, GSA-0920 and GSA-1202, all solvents and reagents were purchased from commercial sources and were of the highest grade available unless otherwise noted. Flash chromatography was performed with silica gel (230/400 mesh, Fisher Scientific) on Biotage Sp1 purification system. All anhydrous reactions were carried out under positive pressure of nitrogen or argon. All microwave reactions were performed on Biotage microwave initiator 2.5. HPLC-MS analyses were performed on Agilent 1100 series instrument with Zorbax C18 reverse phase column unless otherwise noted. HRMS results were obtained on an apex-Qe instrument. All ^{1}H-NMR and ^{13}C-NMR spectra were recorded on a Bruker 300 MHz or DRX 500 MHz NMR spectrometer, using deuterated solvents. The spectra are reported in ppm and referenced to deuterated DMSO (2.49 ppm for ^{1}H, 39.5 ppm for ^{13}C) or referenced to deuterated chloroform (7.26 ppm for ^{1}H, 77 ppm for ^{13}C). Details of ^{1}H-NMR and ^{13}C-NMR data, high resolution mass spectral data and purity of GSA-0825, GSA-0903, GSA-0920 and GSA-1202 are included in the supplementary materials.

Synthesis of GQC-05 (NSC338158; 2-((5,11-dimethyl-6*H*-pyrido[4,3-*b*]carbazol-9-yl)oxy)-*N*,*N*-dimethylethan-1-amine), quindoline (N^1,N^1-diethyl-N^2-(10*H*-indolo[3,2-*b*]quinolin-11-yl)ethane-1,2-diamine) and GSA-0820 (2-(4-(10*H*-indolo[3,2-*b*]quinolin-11-yl)piperazin-1-yl)-*N*,*N*-dimethylethan-1-amine), and GSA-0932, GSA-1502, GSA-1504, GSA-1512 have been reported elsewhere (Boddupally et al., 2012; Brown et al., 2011); (Miranti et al., 2020).

2.2 Cell lines and viruses

Human bone osteosarcoma (U-2 OS) cells (ECACC 92022711) were grown in Dulbecco's Modified Eagle Medium (DMEM, Gibco, Thermo Fisher Scientific, Waltham, MA, USA) supplemented with 10% heat-inactivated fetal bovine serum (FBS, Gibco, Thermo Fisher Scientific, Waltham, MA, USA). Cells were maintained in a humidified incubator set at 37 °C with 5% CO_2. HSV-1 strain F was a kind gift of Professor B. Roizman (University of Chicago, Illinois, USA). The recombinant HSV-1 expressing VP16-GFP (HSV-1 v41) virus (La Boissière et al., 2004) was kindly provided by Professor P. O'Hare (Imperial College London, London, UK).

2.3 Antiviral assay

The antiviral activity of the tested compounds was investigated by plaque reduction assay (PRA) (Blaho et al., 2006).For virus infection, wild- type (wt) HSV-1 (strain F) was added to 24 h plated U-2 OS cells at a multiplicity of infection (MOI) of 1 in serum-free medium. After 1h at 37 °C, the inoculums were replaced with complete medium, and compounds were next added at increasing concentrations (from 25 to 400 nM). Since a single round of HSV-1 replication takes around 24 h to complete, supernatants were collected 24 hours post-infection (hpi.), and stored at -80 °C until viral titrations (Lehman and Boehmer, 1999). For PRA, 24h seeded U-2 OS cells (80000 cells/well in 24 well plates) were infected with serially diluted (10-folds) supernatants for 1h at 37 °C. After infection, cells were washed with 1X PBS and incubated with 500 µL of DMEM supplemented with 0.6% methylcellulose (Sigma-Aldrich, Milan, Italy) and 2% FBS. At 48 hpi, cells were washed with 1X PBS and fixed with formaldehyde 5% in 1X PBS for 20 min at RT, then colored with crystal violet 0.6% (in 20% ethanol). Viral plaques were counted using an optical microscope (Zeiss, Jena, Germany). Data were analyzed by the Reed–Muench method (Lei et al., 2020).

2.4 Time-of-addition (TOA) assay

The time of addition assay was employed to establish which is the last step of the viral life cycle affected by the presence of a compound, as reported by (Daelemans et al., 2011). U-2 OS cells were seeded in 24-well plates at a density of 80000 cells/well and incubated overnight. The day after, cells were infected with HSV-1 strain F at an MOI of 1 and treated every two hours (from 0 to 10hpi) with the tested compound (12.375 µM, corresponding to 75-fold its IC_{50}) or with ACV (45 µM, corresponding to 75-fold its IC_{50}) as reference drug. The supernatants were collected at 24 hpi, and then titrated in Vero CCL81 (African green monkey kidney, 84113001, Sigma Aldich, Milan, Italy) following the PRA working protocol described above.

2.5 Cytotoxicity assay

The cytotoxicity of the tested compounds was evaluated at the same time of antivirals by the MTT assay (Sigma-Aldrich, Milan, Italy). U-2 OS cells were plated into 96-microwell plates (9000 cells/well) to a final volume of 100 µL. The following day, serial dilutions of compounds (from 25 µM to 100 µM) were added to each well and tested in triplicate. Control cells were treated in the exact same conditions. Twenty-four hours

after treatments, cells were supplemented with 10 µL of freshly dissolved solution of MTT (5 mg/mL in PBS 1X) to each well and incubated for 4 h at 37 °C. MTT crystals were then solubilized in solubilization solution (10% sodium dodecyl sulfate (SDS) and 0.01M HCl). After overnight incubation at 37 °C, absorbance was read by Sunrise Tecan plate reader (Mannendorf, Switzerland) at 540 nm. Data were expressed as mean values of at least three individual experiments conducted in triplicate. The percentage of cell survival was calculated as follows: cell survival = $(A_{well} - A_{blank})/(A_{control} - A_{blank}) \times 100$, where blank denotes the medium without cells. Each experiment was repeated at least three times. Data were analyzed by the Reed–Muench method (Lei et al., 2020).

2.6 Oligonucleotides and compounds

All oligonucleotides used in this study were purchased from Sigma-Aldrich (Milan, Italy) and are listed in Table S1. The control compound acyclovir (ACV) was purchased from Sigma-Aldrich (Merck Life Science, Milan, Italy).

2.7 Circular dichroism (CD) analysis

The oligonucleotides were diluted to a final concentration of 4 µM in 10 mM lithium cacodylate buffer supplemented with different concentrations of KCl (2.5 and 100 mM). All samples were denatured at 95 °C for 5 min and gradually cooled to room temperature. Where specified, compounds were added at a final concentration of 16 µM. CD spectra were recorded on a Chirascan-Plus (Applied Photophysics, Leatherhead, UK) equipped with a Peltier temperature controller using a quartz cell of 5 mm path length, over a wavelength range of 230-320 nm. Thermal unfolding analyses were performed over a temperature range of 20–90 °C, with temperature increase of 5 °C. The reported spectra are baseline-corrected for buffer contribution. Observed ellipticities were converted to mean residue ellipticity $(\theta) = deg \times cm^2 \times dmol^{-1}$ *(mol ellip)*. T_m values were calculated according to the van ’t Hoff equation, applied for a two-state transition, assuming that the heat capacity of the folded and unfolded states are equal (Nj, 2006).

2.8 Taq Polymerase stop assay

The DNA primer (Table S1) was 5’-end-labeled with [γ-^{32}P-ATP] using T4 polynucleotide kinase (Thermo Scientific, Milan, Italy) at 37 °C for 30 min and then purified with Illustra MicroSpin G-25 columns (GE Healthcare, Milan, Italy). The labeled primer (final concentration 72 nM) was annealed to the templates (final concentration 36 nM) in lithium cacodylate buffer (10 mM, pH 7.4) in the presence or absence of the indicated concentration of KCl, by heating at 95°C for 5 min and gradually cooling to RT to allow both primer annealing and G4 folding. Where indicated, samples were incubated with various concentrations (2-8 µM) of the selected compound (GSA-0932) at RT overnight. For primer extension, AmpliTaq Gold DNA polymerase (2U/reaction, Applied Biosystems, Carlsbad, California, USA) was incubated at the indicated temperature for 30 min. Reactions were stopped by ethanol precipitation, and primer extension products were separated on a 16% denaturing gel, finally visualized by phosphorimaging (Typhoon FLA 9000, GE Healthcare, Milan, Italy).

2.9 Mass spectrometry (MS) competition assay

Oligonucleotides were heat-denatured and folded in 0.4 mM KCl, 120 mM trimethylammonium acetate (TMAA), pH 7.4, and 20 % isopropanol (IPA) overnight at 4 °C. The oligonucleotides were diluted to a final concentration of 4 μM and incubated with the tested compound at ratio DNA:compound 1:1.5 overnight at 4 °C. Samples were analyzed by direct infusion electrospray ionization (ESI) on a Xevo G2-XS QTof mass spectrometer (Waters, Manchester, UK). The injection was automatically performed by an Acquity UPLC h-class (Waters) equipped with an auto sampler; the carrying buffer was TMAA 120 mM, pH 7.4, 20% IPA. Up to 5 μL samples were typically injected for each analysis. The ESI source settings were the following: electrospray capillary voltage set at 1.8 kV, the source and desolvation temperatures were 45°C and 65°C respectively, the sampling cone was at set at 65V. All these parameters ensured minimal fragmentation of the DNAs complexes. The instrument was calibrated using a 2 mg/mL solution of sodium iodide in 50% of IPA. Binding affinities were calculated for each experiment using the peak intensity for each species calculated by MassLynx V4.1. The binding affinity was calculated with the following formula: [$BA = (\Sigma G4b/(\Sigma G4f + \Sigma G4b)) \times 100$], where BA is the binding affinity, G4b is the intensity of bound G4 DNA, and G4f is the intensity of free G4 DNA.

2.10 UV-visible and fluorescence spectra

Spectrophotometric measures were performed using a Lambda 25 UV-Vis spectrophotometer (Perkin Elmer, Milan, Italy). Absorption spectra and determination of maximum absorption wavelength were carried out in 10 mM lithium cacodylate buffer, pH 7.4 at a compound concentration of 25 μM. The emission spectra were performed on an LS-55 fluorescence spectrophotometer (Perkin Elmer, Milan, Italy) at a 2.5 μM compound concentration in 10 mM lithium cacodylate buffer, pH7. All instruments were equipped with Peltier temperature controllers. Quartz cuvettes of 0.4-10 cm path length were used.

2.11 Confocal microscopy analysis

U-2 OS cells were seeded at 40000 cells/well in 48-well plates (sterile 48-well glass coverslips) and grown overnight at 37 °C. Cells were next infected with the GFP-expressing recombinant virus v41[25] at MOIs of 3 for 1h at 37°C in serum-free medium. Then, cells were washed with PBS 1X and incubated at 37 C in complete medium for the indicated time (6, 8, 10 hpi). Cells were treated with the GSA-0932 compound (1-6 μM) for 2h at 37°C, as replication inhibitor control. At the appropriate times post infection, cells were washed with PBS 1X and fixed with 2% paraformaldehyde (PFA, Sigma-Aldrich) in PBS 1X for 20 min at RT. Mock-infected cells were treated in the same way as infected cells in each type of experiment and staining. Coverslips were mounted in Vectashield mounting medium (DBA Italia, Milan, Italy). GFP fluorescence was evaluated in the 500-550 nm emission filter range, using an excitation wavelength of 488 nm. For overall fluorescence intensity reduction quantification, cells were counted using ImageJ software, and signals were appropriately normalized. At least three pictures per condition were considered. Images were captured with a Leica TCS SP5 confocal laser scanning microscope (Leica microsystems, Germany). Laser excitation and emission filters were: diode

laser λ_{ex} at 405 nm, λ_{em} 415-460 nm, in order to visualize the GSA-0932 fluorescence; whereas blue argon laser λ_{ex} at 488 nm, λ_{em} 500–550 nm, to detect the GFP fluorophore. Final images included in this work are representative of multiple experiments.

2.12 Immunoblot assay

U-2 OS cells were seeded at 280000 cells/well in 6-well plates and grown overnight at 37 °C. Cells were infected with the HSV-1 strain F virus, at an MOI of 1. Infection medium was removed 45 min post-infection, and fresh medium containing the GSA-0932 or ACV compounds at increasing concentrations (0-10 µM) was added to the cell monolayer. 6 hpi the cells were harvested and lysated in RIPA buffer (20 mM Tris-HCl pH 7.5, 150 mM NaCl, 1 mM EGTA, 1% IGEPAL® CA-630, 1% sodium deoxycholate, 1 mM Na_3VO_4, 1X protease inhibitor cocktail; 25 µl/pellet) for 30min at -20°C. After centrifugation at 13000 rpm for 5 min at 4 C, supernatants were collected, and protein concentrations were quantified by using the Pierce® BCA Protein Assay Kit (Thermo Fisher Scientific, Monza, Italy). Each sample was electrophoresed on 8% SDS-PAGE and transferred to a nitrocellulose blotting membrane (Amersham™ Protan™, GE Healthcare, Milan, Italy) by using trans-blot SD semi-dry transfer cell (Bio-Rad Laboratories, Milan, Italy). The membranes were blocked with 2.5% skim milk in PBS 1X. Membranes were then incubated for 2 h with the respective primary antibody directed against ICP4 (mouse monoclonal, H943, Santa Cruz Biotechnology, CA, USA) and alpha-tubulin (mouse monoclonal; Sigma-Aldrich). After three washes in PBST (0.05% Tween-20 in 1X PBS), membranes were incubated for 1h with ECL Plex Goat-α-Mouse IgG-Cy5 (GE Healthcare, Milan, Italy). Images were captured on the Typhoon FLA 9000 (GE Healthcare, Milan, Italy) and quantified by ImageJ software.

2.13 Real Time PCR

At 6 hours post infection, infected U-2 OS cells were harvested and total RNA purified with Total RNA Purification Kit (NorgenBiotek, Canada, Cat #48400), following manufacturer's instruction. Total RNA was quantified using Nanodrop 2000 (Thermo Scientific). Each PCR mixture contained 60 ng of extracted RNA, 200 nM concentrations of both the forward and the reverse primer, and 300 nM of th probe. RT-PCR analysis was carried out in a Lightcycler 480 (Roche, Milan, Italy) with TaqPath™ 1-Step RT-qPCR Master Mix, CG (Thermo Scientific, Cat #A15300).under the following conditions: retrotranscription step (10 min at 50°C), and denaturation step (10 min at 95°C), followed by 45 cycles of 15 s at 95°C and 1 min at 60°C. ICP4 specific primers and probes were designed for this study, whereas Tata Box Binding protein (TBP) primers and probes were from Radonic et al (Radonić et al., 2005). Primers for qPCR are listed in Supplementary Table 1. Each PCR run contained negative controls. Each experiment was conducted in duplicated and two biological replicates were performed. Results were analyzed according to the $2^{-\Delta\Delta CT}$ method (Livak and Schmittgen, 2001).

Table S1. Oligonucleotides used in the study.

**Assays**	**Name**	**Sequence (5'-3')**
CD, MS analysis	un2	GGGGGCGAGGGGCGGGAGGGGGCGAGGGG
	un3	GGGAGGAGCGGGGGGAGGAGCGGG
	gp054a	GGGGTTGGGGCTGGGGTTGGGG
	hTel21	GGGTTAGGGTTAGGGTTAGGG
	c-myc	TGGGGAGGGTGGGGAGGGTGGGGAAGG
	ICP4-146532	GGGCGGGGCGCGAGGGCGGGTGGG
	ICP4-146666	GGGGTGGCCCGCCGGGGGGGCGGGGGG
	ICP4-146574-8	GGGCGGGGCCGGGGGTTCGACCAACGGGCCGCGGCCACGGG
	ICP4-146947	GGCGGGGGTCGTCGGGGTCCGTGGG
Taq pol stop assay	*Taq* primer	GGCAAAAAGCAGCTGCTTATATGCAG
	un2	TTTTTGGGGGCGAGGGGCGGGAGGGGGCGAGG GGTTTTTCTGCATATAAGCAGCTGCTTTTTGCC
	un3	TTTTTGGGAGGAGCGGGGGGAGGAGCGGGTTTT TCTGCATATAAGCAGCTGCTTTTTGCC
	gp054a	TTTTTGGGGTTGGGGCTGGGGTTGGGGTTTTTCT GCATATAAGCAGCTGCTTTTTGCC
	HSV *CTR no* G4	TTGTCGTTAAAGTCTGACTGCGAGCTCTCAGATCC TGCATATAAGCAGCTGCTTTTTGCC
RT-PCR	ICP4 fwd	CGGTGATGAAGGAGCTGCTGTTGC
	ICP4 rev	CTGATCACGCGGCTGCTGTACA
	ICP4 probe	6-FAM-CTGGTCCAGCGCCACGTC-TAMRA
	TBP fwd	TTCGGAGAGTTCTGGGATTGTA
	TBP rev	TGGACTGTTCTTCACTCTTGGC
	TBP probe	6-FAM-CCGTGGTTCGTGGCTCTCTTATCCTCAT-TAMRA

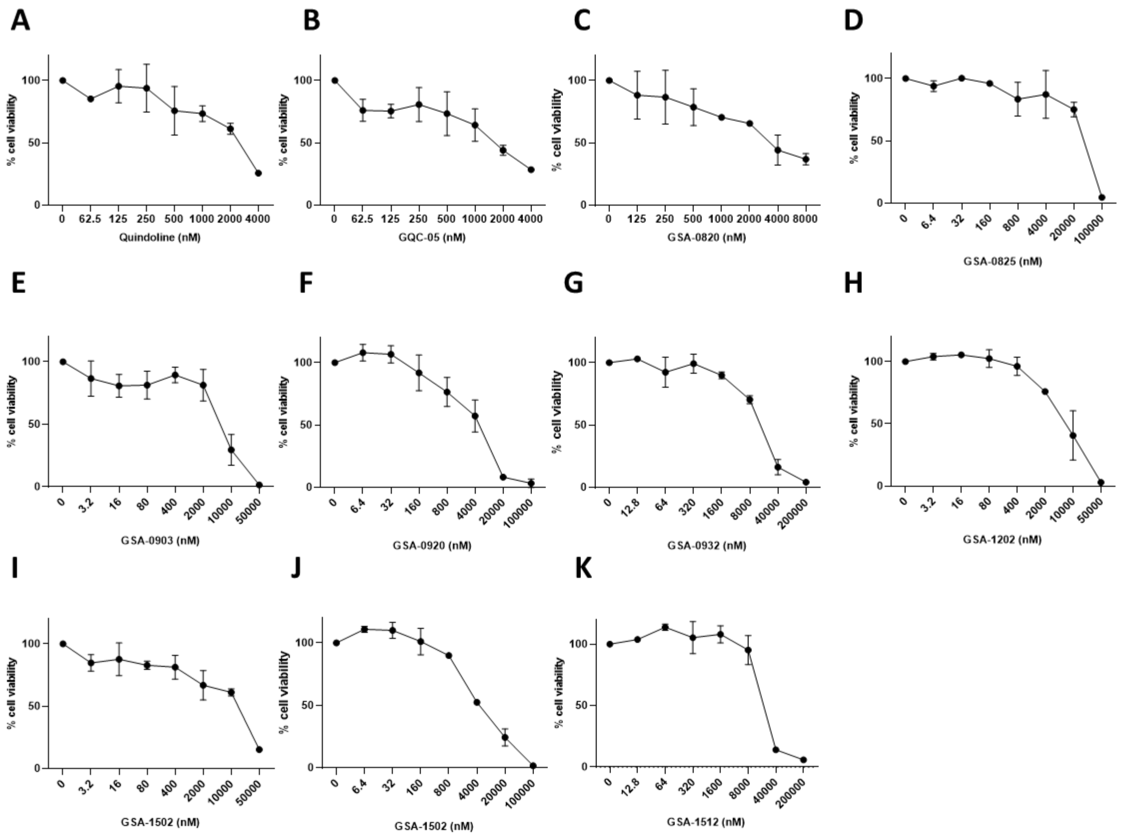

Fig. S1. Antiproliferative activity of Quindoline and derivatives in U-2 OS cells

U-2 OS cells were seeded and treated with the Quindoline and derivatives at the indicated concentrations for 48 h. Cell viability was measured by MTT assay. Mean values of two independent experiments with three replicates per condition and SD are reported.

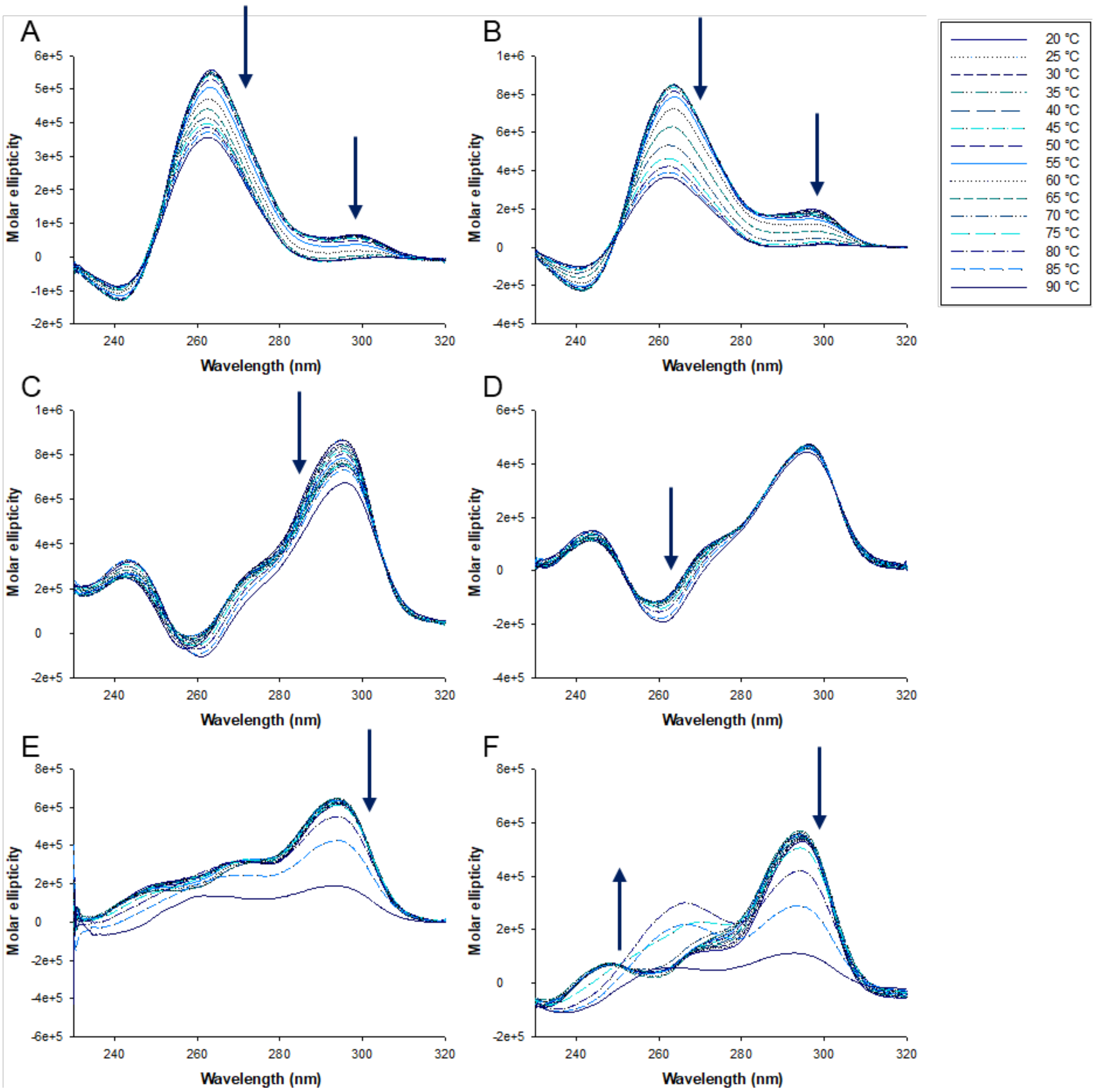

Fig. S2. Thermal unfolding of the HSV-1 G4 sequences folded in 100 mM KCl in the absence and in the presence of GSA-0932. (A) un3 G4 alone and (B) in the presence of GSA-0932; (C) un2 G4 alone and (D) with GSA-0932; (E) gp054a G4 alone and (F) with GSA-0932. Spectra were recorded over a temperature range of 20-90 °C. Oligonucleotide folding was tested in two independent assays, one replicate per condition. The figure shows spectra of one measurement per oligonucleotide. The legend showing spectra color lines coupled with CD temperatures is reported. Arrows indicate the direction of changes in molar ellipticity.

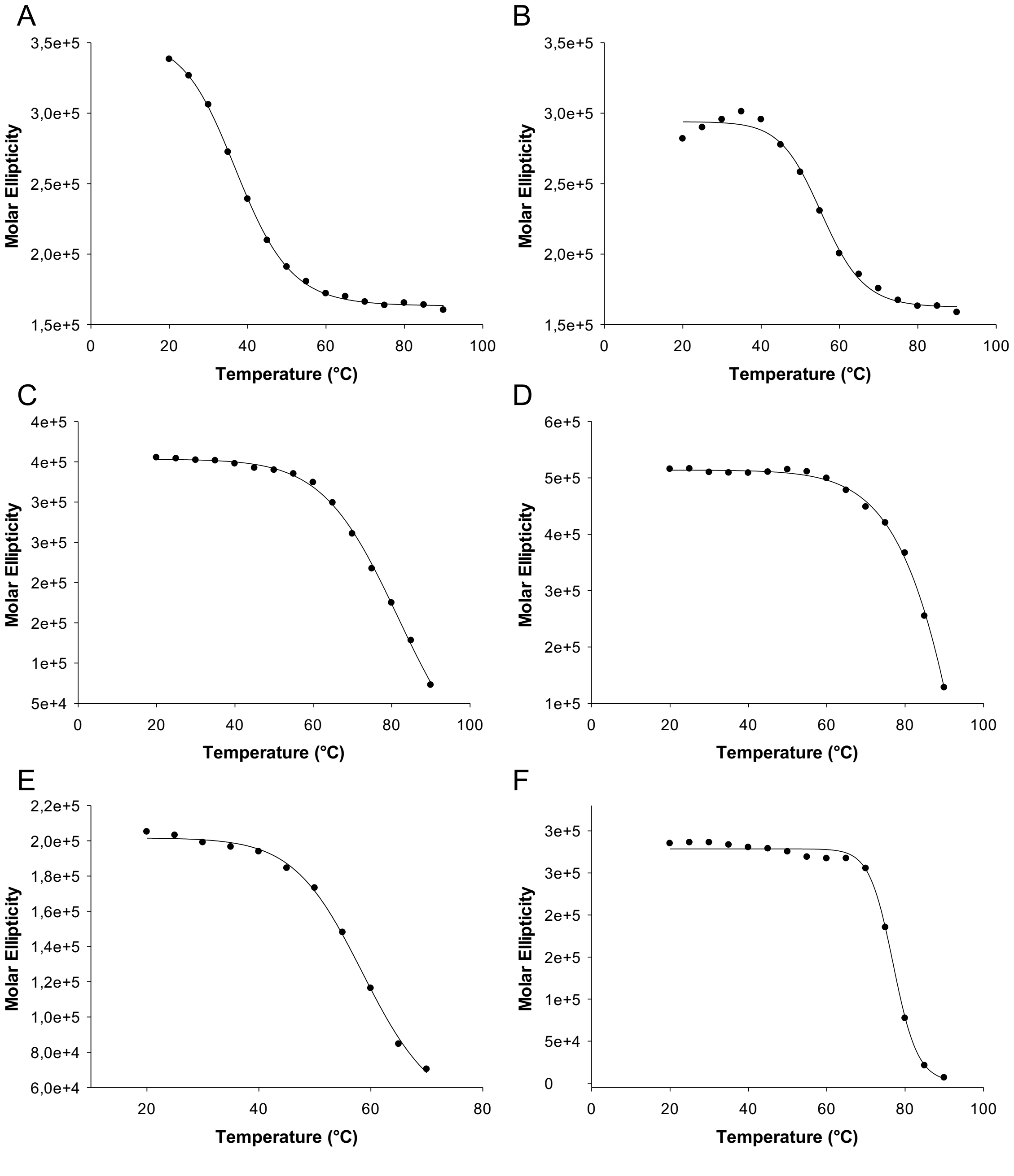

Figure S3: Representative thermal unfolding profiles of HSV-1 G4 sequences in the absence and presence of GSA-0932. (A) un3 G4 alone and (B) with GSA-0932; (C) un2 G4 alone and (D) with GSA-0932; (E) gp054a G4 alone and (F) with GSA-0932. Oligonucleotides were folded in the presence of 2.5 mM KCl; spectra were recorded over a temperature range of 20-90 °C. Oligonucleotide folding was tested in two independent assays, one replicate per condition. The figure shows spectra of one measurement per oligonucleotide.

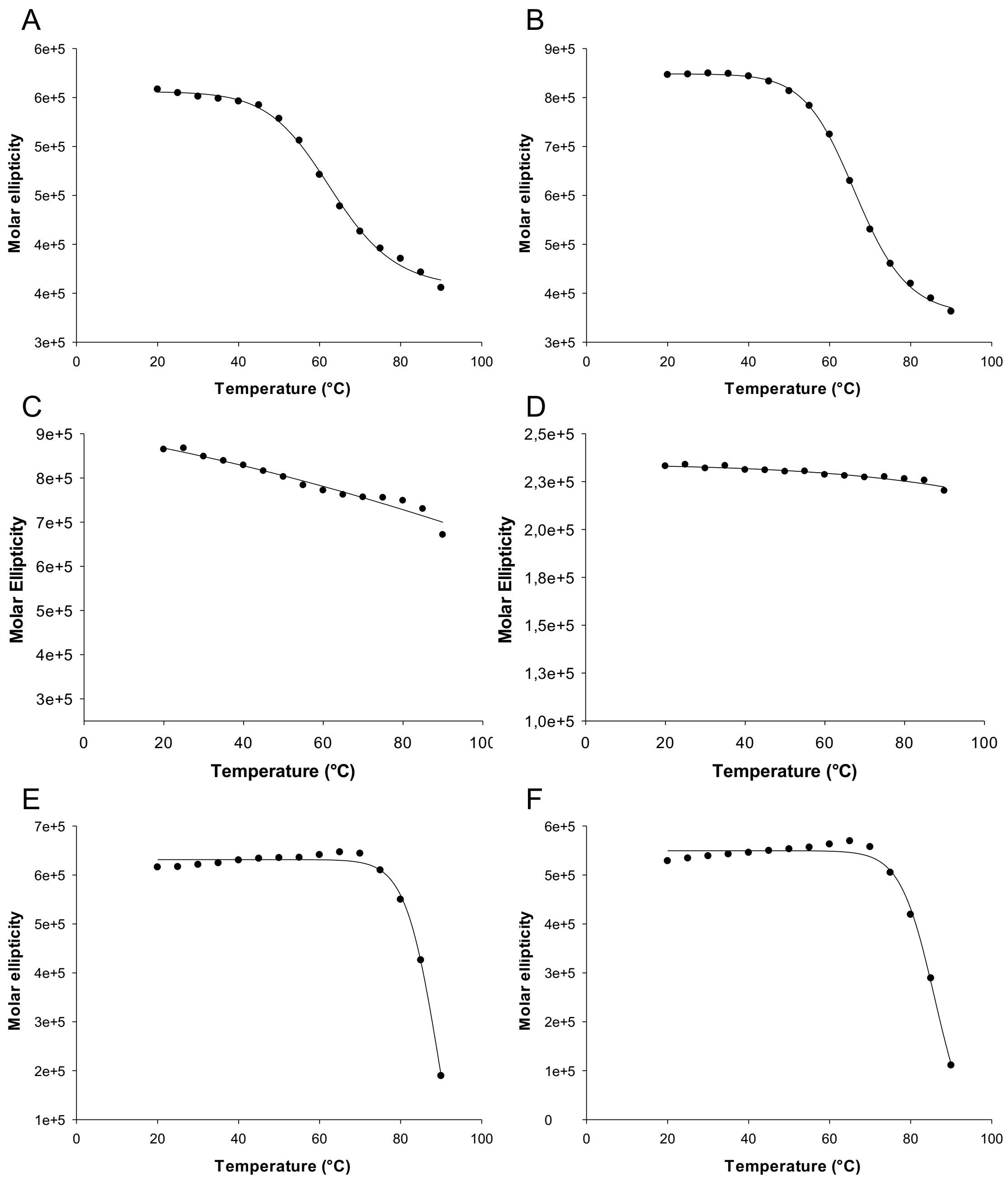

Figure S4: Representative thermal unfolding profiles of HSV-1 G4 sequences in the absence and presence of GSA-0932. (A) un3 G4 alone and (B) with GSA-0932; (C) un2 G4 alone and (D) with GSA-0932; (E) gp054a G4 alone and (F) with GSA-0932. Oligonucleotides were folded in the presence of 100 mM KCl; spectra were recorded over a temperature range of 20-90 °C. Oligonucleotide folding was tested in two independent assays, one replicate per condition. The figure shows spectra of one measurement per oligonucleotide.

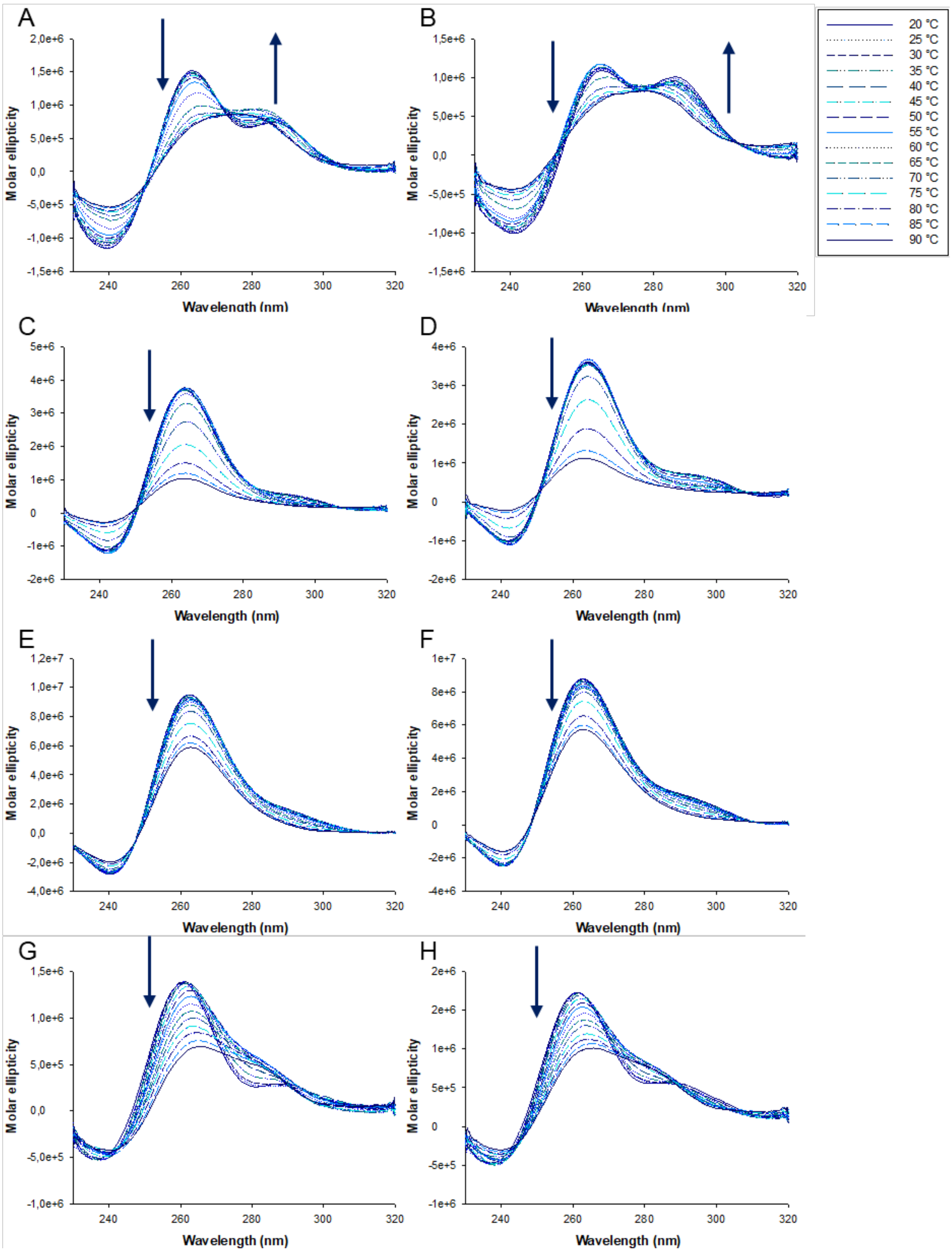

Fig. S5. Thermal unfolding of the HSV-1 Immediate Early promoter G4 sequences in the absence and in the presence of GSA-0932. (A) ICP4 146574-78 G4 alone and (B) in the presence of GSA-0932; (C) ICP4 146532G4 alone and (D) with GSA-0932; (E) ICP4 146666 G4 alone and (F) with GSA-0932; (G) ICP4 146947 G4 alone and (H) with GSA-0932. Oligonucleotides were folded in presence of 2.5 mM KCl and spectra were recorded over a temperature range of 20-90 °C. Oligonucleotide folding was tested in two independent assays, one replicate per condition. The figure shows spectra of one measurement per oligonucleotide. The legend

showing spectra color lines coupled with CD temperatures is reported. Arrows indicate the direction of changes in molar ellipticity.

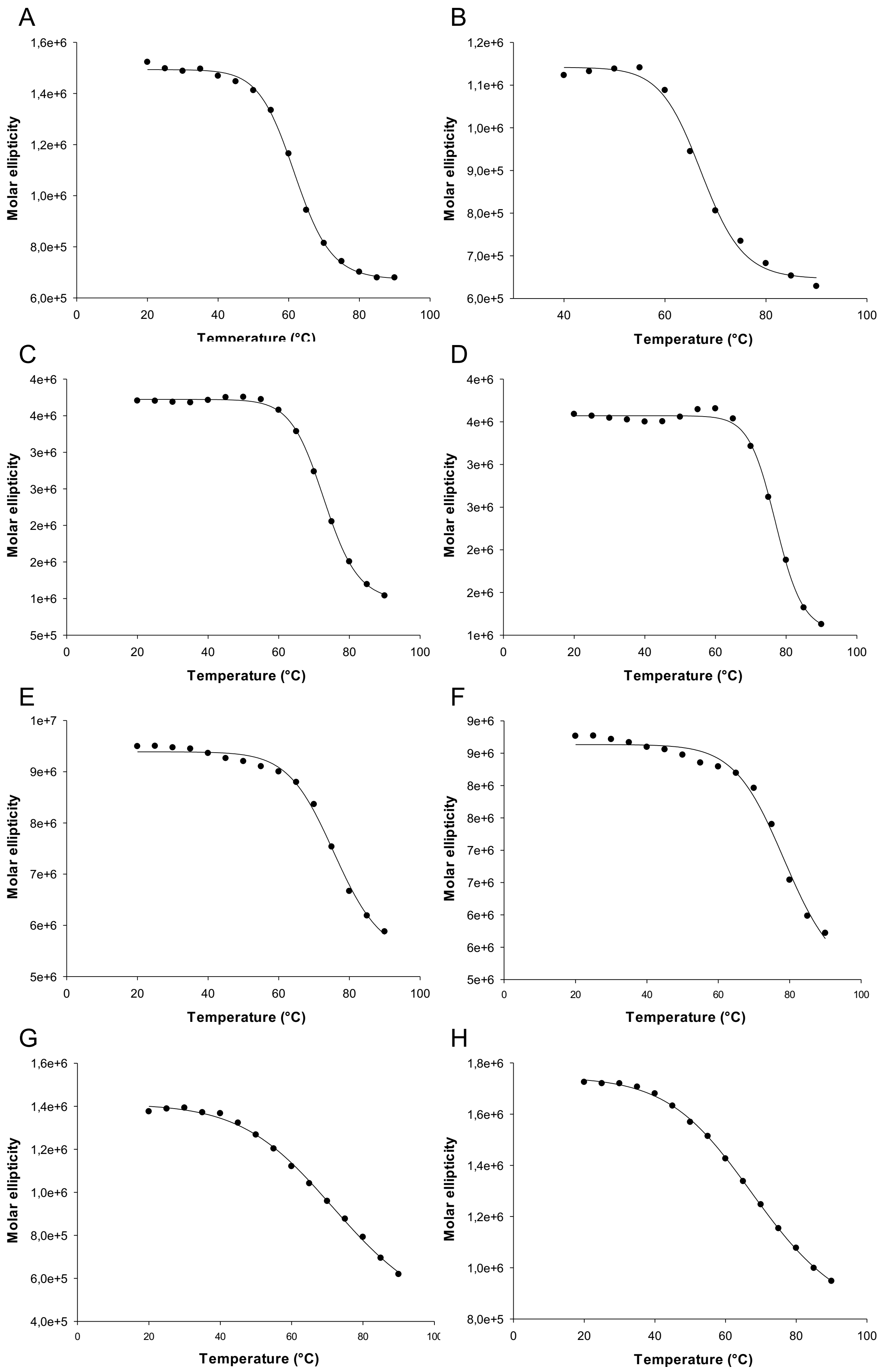

A
1,6e+6
1,4e+6
1,2e+6
1,0e+6
8,0e+5
6,0e+5
Molar ellipticity
0
20
40
60
80
100
B
1,2e+6
1,1e+6
1,0e+6
9,0e+5
8,0e+5
7,0e+5
6,0e+5
Molar ellipticity
40
60
80
100
Temperature (°C)
C
4e+6
3e+6
2e+6
1e+6
5e+5
Molar ellipticity
Temperature (°C)
D
4e+6
3e+6
2e+6
1e+6
Molar ellipticity
Temperature (°C)
E
1e+7
9e+6
8e+6
7e+6
6e+6
5e+6
Molar ellipticity
Temperature (°C)
F
9e+6
8e+6
7e+6
6e+6
5e+6
Molar ellipticity
Temperature (°C)
G
1,6e+6
1,4e+6
1,2e+6
1,0e+6
8,0e+5
6,0e+5
4,0e+5
Molar ellipticity
Temperature (°C)
H
1,8e+6
1,6e+6
1,4e+6
1,2e+6
1,0e+6
8,0e+5
Molar ellipticity
Temperature (°C)

Figure S6: Representative thermal unfolding profiles of HSV-1 G4 sequences in the absence and presence of GSA-0932. (A) ICP4 146574-78 G4 alone and (B) with GSA-0932; (C) ICP4 146532 G4 alone and (D) with GSA-0932; (E) ICP4 146666 G4 alone and (F) with GSA-0932; (G) ICP4 146947 G4 alone and (H) with GSA-0932. Oligonucleotides were folded in the presence of 2.5 mM KCl; spectra were recorded over a temperature range of 20-90 °C. Oligonucleotide folding was tested in two independent assays, one replicate per condition. The figure shows melting profiles of one measurement per oligonucleotide.

Table S2. Melting temperatures (T_m) of ICP4 promoter G4 sequences in the absence/presence of GSA-0932. Oligonucleotides were folded in presence of 2.5 mM KCl. T_m values (°C) were calculated according to the van 't Hoff equation. SD indicates standard deviation.

	**T_m (°C)**	**ΔT_m ± SD (°C)**
ICP4 146574-78	61.8±0.3	
ICP4 146574-78+GSA-0932	67.3±0.6	5.5±0.5
ICP4 146532	72.9±0.2	
ICP4 146532+GSA-0932	77.1±0.4	4.2±0.3
ICP4 146666	76.0±1.1	
ICP4 146666+GSA-0932	78.9±1.2	2.9±1.1
ICP4 146947	69.0±0.7	
ICP4 146947+GSA-0932	73.4±2.0	4.4±1.4

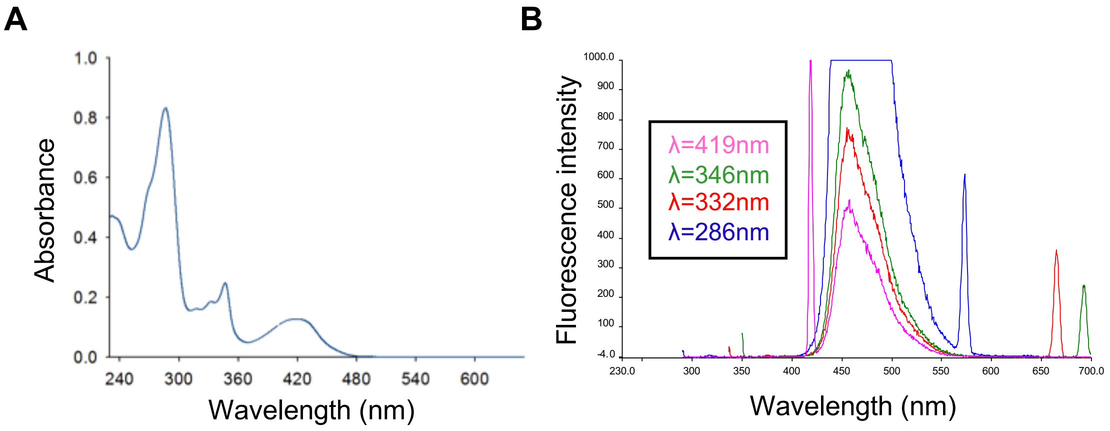

Fig. S7. Spectroscopic properties of GSA-0932. (**A**) UV-visible spectra were carried out in 10 mM lithium cacodylate buffer, pH 7.4 at a compound concentration of 25 μM using a Lambda 25 UV-Vis spectrophotometer (Perkin Elmer). (**B**) Emission spectra were performed on an LS-55 fluorescence spectrophotometer (Perkin Elmer) at a 2.5 μM compound concentration in 10 mM lithium cacodylate buffer, pH 7.4.

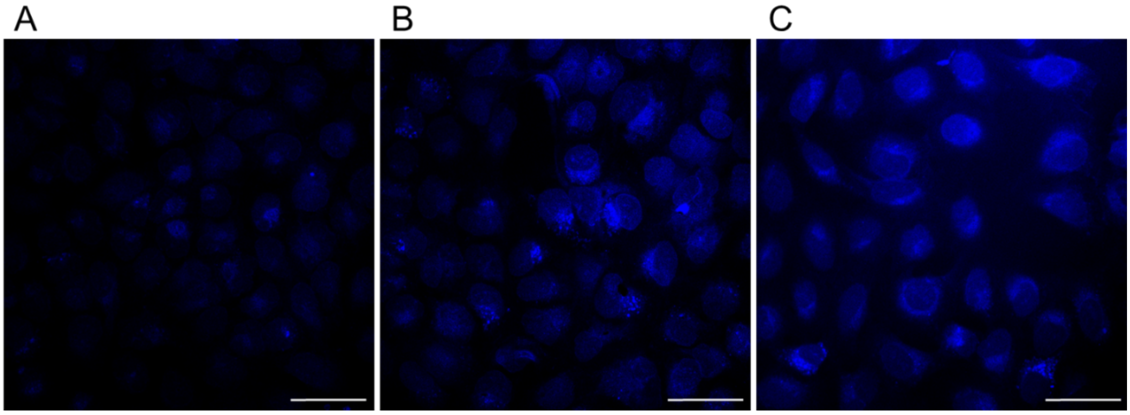

Fig. S8. GSA-0932 distribution in U-2 OS cells

U-2 OS cells were seeded on coverslips and treated for 2 h with the tested compound. (A) GSA-0932 (4 μM), (B) GSA-0932 (6 μM) (C) GSA-0932 (8 μM).

Analytical data on quindoline analogs

GSA-0825

^{1}H NMR (300 MHz, $CDCl_3$): δ 8.53 (d, *J* = 7.5 Hz, 1H, ArH), 8.32-8.23 (m, 2H, ArH), 7.70-7.58 (m, 2H, ArH), 7.54-7.40 (m, 2H, ArH), 7.33 (t, *J* = 7.35 Hz, 1H, ArH), 4.23 (t, *J* = 5.7 Hz, 2H), 3.78-3.60 (m, 6H), 3.43 (t, *J* = 7.65 Hz, 2H), 2.51-2.32 (m, 8H), 2.10-1.94 (m, 2H).

^{13}C NMR (75 MHz, $CDCl_3$): δ 147.89, 146.28, 145.88, 139.72, 129.84, 129.74, 129.66, 127.06, 124.37, 124.31, 123.33, 122.48, 120.49, 110.12, 67.34, 56.38, 54.13, 52.76, 50.45 47.14, 26.19, 26.12. MS (ESI):

HRMS = 401.2331, calculated for $[M+H]^+$ 401.2341.

HPLC purity: 100 %.

GSA-0903

^{1}H NMR (300 MHz, $CDCl_3$): δ 8.50 (d, *J* = 7.8 Hz, 1H, ArH), 8.30-8.20 (m, 2H,ArH), 7.68-7.55 (m, 2H, ArH), 7.53-7.38 (m, 2H, ArH), 7.31 (t, *J* = 7.3 Hz, 1H, ArH), 4.20 (t, *J* = 5.7 Hz, 2H), 3.72-3.62 (m, 2H), 3.36 (t, *J* = 7.95 Hz, 2H), 2.65-2.30 (m, 12H), 2.28 (s, 3H), 2.10-1.90 (m, 2H).
^{13}C NMR (75 MHz, $CDCl_3$): 148.11, 146.44, 145.89, 139.62, 129.83, 129.77, 126.96, 124.37, 123.50, 123.45, 122.39, 120.43, 110.11, 55.99, 55.49, 53.61, 52.75, 50.29, 47.12, 46.45, 26.48, 26.07. MS (ESI): *m/z* 414.5 [100%, $(M+H)^+$].
HRMS = 414.2652. calculated for $[M+H]^+$ 414.5658.
HPLC MS purity: 100 %.

GSA-0920

^{1}H NMR (300 MHz, D_2O): δ 7.69 (d, *J* = 8.1 Hz, 1H, ArH), 7.62-7.51 (m, 2H, ArH), 7.46 (d, *J* = 8.1 Hz, 1H, ArH), 7.38-7.18 (m, 2H, ArH), 7.06 (d, *J* = 7.8 Hz, 1H, ArH), 6.97-6.82 (m, 1H, ArH), 3.92-3.75 (m, 2H), 3.68-3.46 (m, 4H), 3.30-3.15 (m, 2H), 3.05-2.88 (m, 2H), 2.87-2.70 (m, 2H), 2.25-1.95 (m, 4H), 1.80-1.58 (m, 4H), 1.57-1.48 (m, 4H).
^{13}C NMR (75 MHz, D_2O): 148.25, 143.26, 135.63, 133.47, 132.12, 132.04, 126.26, 124.32, 122.79, 121.31, 121.22, 118.66, 117.37, 113.00, 111.05, 55.00, 54.67, 54.13, 52.22, 46.99, 26.47, 25.81, 23.71, 23.56. MS (ESI): *m/z* 413.3 [100%, $(M+H)^+$].
HPLC Purity = 100 %.
HRMS = 413.27016, calculated for $[M+H]^+$ 413.2705

GSA-1202

^{1}H NMR (300 MHz, $CDCl_3$): δ 8.52 (d, J = 7.5 Hz, 1H), 8.32 - 8.21 (m, 2H), 7.70 - 7.56 (m, 2H), 7.55 - 7.45 (m, 1H), 7.42 (d, J = 8.1 Hz, 1H), 7.30 (t, J = 7.5 Hz, 1H), 4.15 – 4.28 (m, 2H), 3.72 – 3.65 (m, 2H), 3.35 (t, J = 7.5 Hz, 2H), 2.50 - 2.25 (m, 8H), 2.04 (q, 2H), 1.59 (q, 4H), 1.55 -1.38 (m, 2H).

^{13}C-NMR (75 MHz, $CDCl_3$): δ 148.12, 146.47, 145.87, 139.69, 129.81, 129.79, 129.72, 126.93, 124.40, 124.35, 123.51, 123.48, 122.36, 120.37, 110.10, 56.93, 55.15, 52.87, 50.14, 47.11, 26.66, 26.43, 25.91, 24.86.

HRMS: 399.2543, calculated for $[M+H]^+$ = 399.2543

HPLC Purity = 100 %.

References

Blaho, J.A., Morton, E.R., Yedowitz, J.C., 2006. Herpes Simplex Virus: Propagation, Quantification, and Storage. Current Protocols in Microbiology 00, 14E.1.1-14E.1.23. https://doi.org/10.1002/9780471729259.mc14e01s00

Boddupally, P.V.L., Hahn, S., Beman, C., De, B., Brooks, T.A., Gokhale, V., Hurley, L.H., 2012. The anticancer activity and cellular repression of c-MYC by the G-quadruplex-stabilizing 11-piperazinyl quindoline is not dependent on direct targeting of the G-quadruplex in the c-MYC promoter. J Med Chem 55, 6076–6086. https://doi.org/10.1021/jm300282c

Brown, R.V., Danford, F.L., Gokhale, V., Hurley, L.H., Brooks, T.A., 2011. Demonstration that drug-targeted down-regulation of MYC in non-Hodgkins lymphoma is directly mediated through the promoter G-quadruplex. J Biol Chem 286, 41018–41027. https://doi.org/10.1074/jbc.M111.274720

Daelemans, D., Pauwels, R., De Clercq, E., Pannecouque, C., 2011. A time-of–drug addition approach to target identification of antiviral compounds. Nat Protoc 6, 925–933. https://doi.org/10.1038/nprot.2011.330

La Boissière, S., Izeta, A., Malcomber, S., O'Hare, P., 2004. Compartmentalization of VP16 in Cells Infected with Recombinant Herpes Simplex Virus Expressing VP16-Green Fluorescent Protein Fusion Proteins. J Virol 78, 8002–8014. https://doi.org/10.1128/JVI.78.15.8002-8014.2004

Lehman, I.R., Boehmer, P.E., 1999. Replication of herpes simplex virus DNA. J Biol Chem 274, 28059–28062. https://doi.org/10.1074/jbc.274.40.28059

Lei, C., Yang, J., Hu, J., Sun, X., 2020. On the Calculation of TCID50 for Quantitation of Virus Infectivity. Virol Sin 36, 141–144. https://doi.org/10.1007/s12250-020-00230-5

Livak, K.J., Schmittgen, T.D., 2001. Analysis of Relative Gene Expression Data Using Real-Time Quantitative PCR and the 2−ΔΔCT Method. Methods 25, 402–408. https://doi.org/10.1006/meth.2001.1262

Miranti, C.K., Moore, S., Kim, Y., Chappeta, V.R., Wu, K., De, B., Gokhale, V., Hurley, L.H., Reyes-Reyes, E.M., 2020. Nucleolin represses transcription of the androgen receptor gene through a G-quadruplex. Oncotarget 11, 1758–1776. https://doi.org/10.18632/oncotarget.27589

Nj, G., 2006. Using circular dichroism collected as a function of temperature to determine the thermodynamics of protein unfolding and binding interactions. Nature protocols 1. https://doi.org/10.1038/nprot.2006.204

Radonić, A., Thulke, S., Bae, H.-G., Müller, M.A., Siegert, W., Nitsche, A., 2005. Reference gene selection for quantitative real-time PCR analysis in virus infected cells: SARS corona virus, Yellow fever virus, Human Herpesvirus-6, Camelpox virus and Cytomegalovirus infections. Virol J 2, 7. https://doi.org/10.1186/1743-422X-2-7
